# Bessel statistical convergence: New concepts and applications in sequence theory

**DOI:** 10.1371/journal.pone.0313273

**Published:** 2024-11-14

**Authors:** Ibrahim S. Ibrahim, Majeed A. Yousif, Pshtiwan Othman Mohammed, Dumitru Baleanu, Ahmad Zeeshan, Mohamed Abdelwahed

**Affiliations:** 1 Department of Mathematics, College of Education, University of Zakho, Zakho, Iraq; 2 Department of Mathematics, College of Education, University of Sulaimani, Sulaimani, Iraq; 3 Department of Computer Science and Mathematics, Lebanese American University, Beirut, Lebanon; 4 Department of Mathematics and Statistics, FBAS, International Islamic University Islamabad, Islamabad, Pakistan; 5 Department of Mathematics, College of Sciences, King Saud University, Riyadh, Saudi Arabia; Jinka University, ETHIOPIA

## Abstract

This research introduces novel concepts in sequence theory, including Bessel convergence, Bessel boundedness, Bessel statistical convergence, and Bessel statistical Cauchy sequences. These concepts establish new inclusion relations and related results within mathematical analysis. Additionally, we extend the first and second Korovkin-type approximation theorems by incorporating Bessel statistical convergence, providing a more robust and comprehensive framework than existing results. The practical implications of these theorems are demonstrated through examples involving the classical Bernstein operator and Fejér convolution operators. This work contributes to the foundational understanding of sequence behavior, with potential applications across various scientific disciplines.

## 1 Introduction

Statistical convergence is a natural extension of the traditional concept of convergence, broadening its applicability in various mathematical contexts. This concept was introduced independently by Fast [1] and Steinhaus [2] in 1951, marking a significant development in the study of sequences. Shortly thereafter, Schoenberg [3] also presented the idea independently, further solidifying its place in mathematical theory.

Over the years, statistical convergence has found numerous applications. Its usefulness has grown, extending into numerous areas in recent times. Notable applications include approximation theory [4–6], summability theory [7–9], measure theory [10], time scale [11–13], Fourier analysis [14] and Banach spaces [15, 16]. These diverse applications underscore the versatility and importance of statistical convergence in modern mathematical research. For additional information, refer to [17–20].

Statistical convergence relies on the concept of natural density for subsets of N. Let Ω be a subset of N. The natural density of Ω, represented as Λ(Ω), is given by

$$\begin{array}{c} \Lambda (\Omega )=\underset{\upsilon \to \infty }{lim}\;\frac{1}{\upsilon }|\{\iota \leq \upsilon :\iota \in \Omega \}|, \end{array}$$

in case the limit exists, where |{*ι* ≤ *υ* : *ι* ∈ Ω}| denotes the count of elements in Ω that are less than or equal to *υ* (see [21]).

A sequence $\left({\bar{T}}_{\rho }\right)$ is said to be statistically convergent (or, *S*-convergent) to the value $T^{*}$ if, for every *ϱ* > 0, the set $\left\{\rho \leq \upsilon :|{\bar{T}}_{\rho }-T^{*}|>\varrho \right\}$ has a natural density zero, that is,

$$\begin{array}{c} \underset{\upsilon \to \infty }{lim}\;\frac{1}{\upsilon }|\{\rho \leq \upsilon :|{\bar{T}}_{\rho }-T^{*}|>\varrho \}|=0. \end{array}$$

In this context, the statistical limit of the sequence $\left({\bar{T}}_{\rho }\right)$ is denoted as $T^{*}$, written as $St-lim\;{\bar{T}}_{\rho }=T^{*}$. Throughout this study, *S* represents the set of all *S*-convergent sequences.

In [22], the difference sequence spaces *c*_0_(Δ), *c*(Δ), and *ℓ*_∞_(Δ) were introduced, defined as follows:

$$\begin{array}{c} c_{0}(\Delta )=\{\bar{T}=({\bar{T}}_{\rho }):\Delta \bar{T}\in c_{0}\}, \end{array}$$

$$\begin{array}{c} c(\Delta )=\{\bar{T}=({\bar{T}}_{\rho }):\Delta \bar{T}\in c\}, \end{array}$$

and

$$\begin{array}{c} \ell_{\infty }(\Delta )=\{\bar{T}=({\bar{T}}_{\rho }):\Delta \bar{T}\in \ell_{\infty }\}, \end{array}$$

where $\Delta \bar{T}=(\Delta {\bar{T}}_{\rho })=({\bar{T}}_{\rho }-{\bar{T}}_{k+1})$ and the symbols *c*_0_, *c*, and *ℓ*_∞_ represent the spaces of null sequences, convergent sequences and bounded sequences, respectively.

Mathematics provides a powerful tool for understanding and solving problems related to circular and cylindrical shapes known as Bessel functions. These functions are named after the German mathematician Friedrich Bessel [23] who first introduced them. In various branches of mathematics, science, and engineering, Bessel functions have been extensively used and applied due to their importance and widespread applicability. There is a rich tapestry of mathematical analysis involved in the study of Bessel functions, including their properties, asymptotic behavior, integral representations, and special cases. Furthermore, Bessel functions are applicable beyond theoretical realms, with practical implications for engineering, physics, and other fields of science. The Bessel function of the first kind $J_{w}(\xi )$ is defined by the following series representation:

$$J_{w}(\xi )=\sum_{u=0}^{\infty }\frac{{\left(-1\right)}^{u}}{\Gamma (u+w+1)u!}{\left(\frac{\xi }{2}\right)}^{2u+w},$$

where *w* is a real number known as the order of the Bessel function and Γ denotes the gamma function (also called Euler’s integral) which is defined for *ξ* > 0 by

$$\Gamma (\xi )=\int_{0}^{\infty }e^{-y}y^{\xi -1}dy.$$

Further applications on of this principle are available in [24–28].

In the realm of sequence spaces and summability theory, significant advancements have been made with traditional concepts of convergence and boundedness. However, the introduction of new convergence methods remains a crucial area for exploration. Notably, Bessel functions have not been previously integrated into these frameworks, presenting a distinct gap in the literature. This research addresses this gap by introducing Bessel convergence, Bessel boundedness, Bessel statistical convergence, and Bessel statistical Cauchy sequences. These concepts are developed to provide a more robust and comprehensive understanding of sequence behavior, particularly within the context of Bessel functions. By pioneering the integration of Bessel functions into these concepts, our research not only fills a significant gap in the existing literature but also paves the way for further studies and applications in mathematical analysis and approximation theory. We will now proceed to outline the primary sections of the paper.

## 2 Bessel statistical convergence

In this section, we present the primary findings of our study. We introduce new definitions, including Bessel statistical convergence, and establish key relationships. Additionally, we provide several theorems, supported by proofs, and discuss related work that underpins our research.

The general sequence space $J_{w}^{m}\left(X\right)$ is defined as follows:

$$\begin{array}{c} J_{w}^{m}(X)=\{\bar{T}=({\bar{T}}_{\rho }):J_{w}^{m}({\bar{T}}_{\rho })\in X\}, \end{array}$$

where X is any sequence space, m∈N, *w* is a real number and

$$J_{w}^{m}({\bar{T}}_{\rho })=\sum_{u=0}^{m}\frac{{\left(-1\right)}^{u}}{\Gamma (u+1+w)u!}{\left(\frac{{\bar{T}}_{\rho +u}}{2}\right)}^{2u+w}.$$

Given that X is a linear space, it follows that $J_{w}^{m}\left(X\right)$ will also be a linear space. Moreover, if X possesses the properties of a Banach space, then $J_{w}^{m}\left(X\right)$ will similarly be a Banach space, defined with an appropriate norm

$${\left\|\bar{T}\right\|}_{J}=\sum_{u=1}^{m}|{\bar{T}}_{u}|+\|J_{w}^{m}(\bar{T})\|.$$

**Theorem 2.1**. *If* X⊂Y, *then* $J_{w}^{m}(X)\subset J_{w}^{m}(Y)$.

*Proof*. Straightforward.

**Theorem 2.2**. *Let* X *be a Banach space, and let* B *be a closed subset of* X. *Then, the space* $J_{w}^{m}\left(B\right)$ *remains closed in the space* $J_{w}^{m}\left(X\right)$.

*Proof*. Since B⊂X, by Theorem 2.1, we have $J_{w}^{m}(B)\subset J_{w}^{m}(X)$. Our next step is to establish that $\bar{J_{w}^{m}(B)}=J_{w}^{m}(\bar{B})$, where $\bar{J_{w}^{m}(B)}$ and $\bar{B}$ symbolize the closures of $J_{w}^{m}(B)$ and B, respectively. Let $\bar{T}=({\bar{T}}_{\rho })\in \bar{J_{w}^{m}(B).}$ Consequently, a sequence $\left({\bar{T}}^{\upsilon }\right)$ can be found in $J_{w}^{m}\left(B\right)$ such that

$${\left\|{\bar{T}}^{\upsilon }-\bar{T}\right\|}_{J}\to 0\;\text{as}\;\upsilon \to \infty .$$

This suggests that

$${\left\|({\bar{T}}_{\rho }^{\upsilon })-({\bar{T}}_{\rho })\right\|}_{J}\to 0\;\text{as}\;\upsilon \to \infty$$

in $J_{w}^{m}(B)$. So,

$$\sum_{u=1}^{m}|{\bar{T}}_{u}^{\upsilon }-{\bar{T}}_{u}|+{\left\|J_{w}^{m}({\bar{T}}_{\rho }^{\upsilon })-J_{w}^{m}({\bar{T}}_{\rho })\right\|}_{J}\to 0\;\text{as}\;\upsilon \to \infty$$

in B. That is, $J_{w}^{m}(\bar{T})\in \bar{B}$. Thus, $\bar{T}\in J_{w}^{m}(\bar{B})$. Conversely, suppose that $\bar{T}\in J_{w}^{m}(\bar{B})$. This implies that $\bar{T}\in \bar{J_{w}^{m}(\bar{B})}$. We know that B is closed, then $\bar{J_{w}^{m}(\bar{B})}=\bar{J_{w}^{m}(B)}$. Therefore, $\bar{T}\in \bar{J_{w}^{m}(B)}$, which confirms that $J_{w}^{m}(B)$ is a closed subset of $J_{w}^{m}(X)$.

From Theorem 2.2, we obtain the following result.

**Corollary 2.1**. *If* X *is a separable space, then* $J_{w}^{m}\left(B\right)$ *is also separable*.

**Definition 2.1**. *A sequence* $\left({\bar{T}}_{\rho }\right)$ *is called Bessel convergent (or,* $J_{w}^{m}$-*convergent) to a number* $T^{*}$ *if for every* *ϱ* > 0, *there exists an integer* $n_{o}=n_{o}\left(\varrho \right)\in N$ *such that*

$$|J_{w}^{m}({\bar{T}}_{\rho })-T^{*}|<\varrho \;for\;all\;\rho \geq n_{o}.$$

*In this case, we write* $J_{w}^{m}-lim{\bar{T}}_{\rho }=T^{*}$. *The class of all* $J_{w}^{m}$-*convergent sequences is denoted by* $c[J_{w}^{m}]$, *defined as*

$$c\left[J_{w}^{m}\right]=\{({\bar{T}}_{\rho }):J_{w}^{m}-lim{\bar{T}}_{\rho }=T^{*}\;for\;some\;number\;T^{*}\}.$$

**Definition 2.2**. *A sequence* $\left({\bar{T}}_{\rho }\right)$ *is called Bessel bounded (or,* $J_{w}^{m}$–*bounded) if there exists a positive constant* $M\in R^{+}$ *such that*

$$|J_{w}^{m}({\bar{T}}_{\rho })|\leq M\;for\;all\;\rho \in N.$$

*The class of all* $J_{w}^{m}$-*bounded sequences is denoted by* $\ell_{\infty }\left[J_{w}^{m}\right]$, *defined as*

$$\ell_{\infty }\left[J_{w}^{m}\right]=\{({\bar{T}}_{\rho }):\exists M\in R^{+}\;with\;|J_{w}^{m}({\bar{T}}_{\rho })|\leq M\;for\;all\;\rho \in N\}.$$

**Definition 2.3**. *A sequence* $\left({\bar{T}}_{\rho }\right)$ *is called Bessel statistically convergent (or*, $S(J_{w}^{m})$-*convergent) to a number* $T^{*}$ *if for every* *ϱ* > 0, *the set* $\left\{\rho \leq \upsilon :|J_{w}^{m}({\bar{T}}_{\rho })-T^{*}|\geq \varrho \right\}$ *has natural density zero. In other words*,

$$\underset{\upsilon \to \infty }{\text{lim}}\;\frac{1}{\upsilon }|\{\rho \leq \upsilon :|J_{w}^{m}({\bar{T}}_{\rho })-T^{*}|\geq \varrho \}|=0.$$

*We denote this by* $St\left(J_{w}^{m}\right)-lim{\bar{T}}_{\rho }=T^{*}$. *The class of all* $S(J_{w}^{m})$-*convergent sequences is denoted by* $S[J_{w}^{m}]$, *defined as*

$$S\left[J_{w}^{m}\right]=\{({\bar{T}}_{\rho }):\forall \varrho >0,\underset{\upsilon \to \infty }{\text{lim}}\;\frac{1}{\upsilon }|\{\rho \leq \upsilon :|J_{w}^{m}({\bar{T}}_{\rho })-T^{*}|\geq \varrho \}|=0\;for\;some\;number\;T^{*}\}.$$

**Theorem 2.3**. *If a sequence* $\left({\bar{T}}_{\rho }\right)$ *is* $J_{w}^{m}$–*convergent, then it is* $S(J_{w}^{m})$–*convergent (to the same limit); however, the converse does not have to be correct, in general*.

*Proof*. Suppose that $\left({\bar{T}}_{\rho }\right)$ is $J_{w}^{m}$-convergent to $T^{*}$. Then, for every *ϱ* > 0, there exists $n_{o}\in N$ such that

$$|J_{v}^{m}({\bar{T}}_{\rho })-T^{*}|<\varrho \;\forall \rho \geq n_{o}.$$

This indicates that the set $\left\{\rho \leq \upsilon :|J_{w}^{m}({\bar{T}}_{\rho })-T^{*}|\geq \varrho \right\}$ is finite for every υ∈N, which consequently entails that

$$\underset{\upsilon \to \infty }{\text{lim}}\;\frac{1}{\upsilon }|\{\rho \leq \upsilon :|J_{w}^{m}({\bar{T}}_{\rho })-T^{*}|\geq \varrho \}|=0.$$

Therefore, $\left({\bar{T}}_{\rho }\right)$ is $S(J_{w}^{m})$–convergent to $T^{*}$.

For the converse part, let us consider a sequence $\left({\bar{T}}_{\rho }\right)$ such that

$$\begin{array}{c} J_{w}^{m}({\bar{T}}_{\rho })=\{\begin{array}{cc} \rho & \text{if}\;\rho =\upsilon^{2}\;\\ 1 & \text{if}\;\rho \neq \upsilon^{2} \end{array}\;\upsilon \in N. \end{array}$$

Let *ϱ* > 0 be given. For each υ∈N, we have

$$|\{\rho \leq \upsilon :|J_{w}^{m}({\bar{T}}_{\rho })-1|\geq \varrho \}|\leq \sqrt{\upsilon }.$$

This implies that

$$\underset{\upsilon \to \infty }{lim}\;\;\frac{1}{\upsilon }|\{\rho \leq \upsilon :|J_{w}^{m}({\bar{T}}_{\rho })-1|\geq \varrho \}|\leq \underset{\upsilon \to \infty }{lim}\;\;\frac{1}{\upsilon }\sqrt{\upsilon }=0.$$

So, $St(J_{w}^{m})-lim{\bar{T}}_{\rho }=1$. However, $\left({\bar{T}}_{\rho }\right)$ is not $J_{w}^{m}$–convergent since $\left({\bar{T}}_{\rho })\notin \ell_{\infty }[J_{w}^{m}\right]$. As a result, $c[J_{w}^{m}]⫋S[J_{w}^{m}]$.

**Theorem 2.4**. *A sequence* $\left({\bar{T}}_{\rho }\right)$ *is said to be* $S(J_{w}^{m})$-*convergent to a number* $T^{*}$ *if and only if there exists a set* Ω⊂N *such that* Λ(Ω) = 0 *and* ${\text{lim}}_{\rho \in N\backslash \Omega }\;J_{w}^{m}({\bar{T}}_{\rho })=T^{*}$.

*Proof*. Let $\Omega^{t}=\{\rho \in N:|J_{w}^{m}({\bar{T}}_{\rho })-T^{*}|>\frac{1}{t}\}$ for t∈N. As $\left({\bar{T}}_{\rho }\right)$ is $S(J_{w}^{m})$–convergent to $T^{*}$ so that Λ(Ω^*t*^) = 0. It is clear that Ω^*t*^ ⊂ Ω^*t*+1^ for each t∈N. We only need to prove the case where some of the Ω^*t*′^s are non-empty. Assume that Ω^1^ ≠ *ϕ*. Take any *e*_1_ ∈ Ω^1^. Next, take *e*_2_ ∈ Ω^2^ such that *e*_2_ > *e*_1_ and

$$\underset{\upsilon \to \infty }{lim}\;\;\frac{1}{\upsilon }|\{\rho \leq \upsilon :|J_{w}^{m}({\bar{T}}_{\rho })-T^{*}|>\frac{1}{2}\}|<\frac{1}{2},$$

for all *υ* ≥ *e*_2_. As a result, we get *e*_1_ < *e*_2_ < *e*_3_ < … with *e*_*t*_ ∈ Ω^*t*^ and

$$\underset{\upsilon \to \infty }{lim}\;\;\frac{1}{\upsilon }|\{\rho \leq \upsilon :|J_{w}^{m}({\bar{T}}_{\rho })-T^{*}|>\frac{1}{t}\}|<\frac{1}{t}$$

for all *υ* ≥ *e*_*t*_. Now, consider Ω = ([*e*_*t*_, *e*_*t*+1_) ∩ Ω^*t*^). Then, $\Omega \subset \Omega^{t}$ for some *t* and leads to

$$\begin{array}{cc} \frac{\left|\Omega \right|}{\upsilon }\leq \frac{\left|\Omega^{t}\right|}{\upsilon } & =\frac{1}{\upsilon }|\{\rho \leq \upsilon :|J_{w}^{m}({\bar{T}}_{\rho })-T^{*}|>\frac{1}{t}\}|\\ & \leq \frac{1}{t}. \end{array}$$

To establish that Λ(Ω) = 0, we proceed as follows. Let *ϱ* > 0 be given. We can select an integer t∈N such that $\frac{1}{t}<\varrho$. For any ρ∈N\Ω with *ρ* ≥ *e*_*t*_, there exists an integer *r* ≥ *t* such that *e*_*r*_ ≤ *ρ* ≤ *e*_*r*+1_, which implies that *ρ* ∉ Ω^*r*^. Thus, we have

$$|J_{w}^{m}({\bar{T}}_{\rho })-T^{*}|<\frac{1}{r}\leq \frac{1}{t}<\varrho .$$

This shows that ${\text{lim}}_{\rho \in N\backslash \Omega }\;J_{w}^{m}({\bar{T}}_{\rho })=T^{*}$.

Conversely, assume there exists a subset Ω⊂N such that Λ(Ω) = 0 and ${\text{lim}}_{\rho \in N\backslash \Omega }\;J_{w}^{m}({\bar{T}}_{\rho })=T^{*}$. Given any *ϱ* > 0, there exists an integer $n_{0}(\varrho )\in N$ such that

$$|J_{w}^{m}({\bar{T}}_{\rho })-T^{*}|<\varrho \;\text{for}\;\text{all}\;\rho \geq n_{0}\;\text{and}\;\rho \notin \Omega .$$

This implies that

$$\{\rho \in N:|J_{w}^{m}({\bar{T}}_{\rho })-T^{*}|>\varrho \}\subset \Omega \cup \{1,2,\ldots ,n_{0}\}.$$

Therefore,

$$\underset{\upsilon \to \infty }{\text{lim}}\;\frac{1}{\upsilon }|\{\rho \leq \upsilon :|J_{w}^{m}({\bar{T}}_{\rho })-T^{*}|\geq \varrho \}|=0.$$

Thus, $\left({\bar{T}}_{\rho }\right)$ is $S(J_{w}^{m})$–convergent to $T^{*}$.

**Theorem 2.5**. *A sequence* $\left({\bar{T}}_{\rho }\right)$ *is* $S(J_{w}^{m})$-*convergent to a number* $T^{*}$ *if and only if there exists a sequence* $\left({\bar{Y}}_{\rho }\right)$ *that is* $J_{w}^{m}$-*convergent to* $T^{*}$ *and*

$$\Lambda (\{\rho \in N:J_{w}^{m}({\bar{T}}_{\rho })\neq J_{w}^{m}({\bar{Y}}_{\rho })\})=0.$$

*Proof*. Assume that the sequence $\left({\bar{T}}_{\rho }\right)$ is $S(J_{w}^{m})$–convergent to $T^{*}$. According to Theorem 2.4, there exists a set Ω⊂N such that Λ(Ω) = 0 and ${\text{lim}}_{\rho \in N\backslash \Omega }\;J_{w}^{m}({\bar{T}}_{\rho })=T^{*}$. We define a new sequence $\left({\bar{Y}}_{\rho }\right)$ such that

$$J_{w}^{m}({\bar{Y}}_{\rho })=\{\begin{array}{cc} T^{*}, & \text{if}\;\rho \in \Omega ,\\ J_{w}^{m}({\bar{T}}_{\rho }), & \text{if}\;\rho \in N\backslash \Omega . \end{array}$$

Then,

$$J_{w}^{m}({\bar{Y}}_{\rho })-T^{*}=\{\begin{array}{cc} 0, & \text{if}\;\rho \in \Omega ,\\ J_{w}^{m}({\bar{T}}_{\rho })-T^{*}, & \text{if}\;\rho \in N\backslash \Omega . \end{array}$$

Since ${\text{lim}}_{\rho \in N\backslash \Omega }\;J_{w}^{m}({\bar{T}}_{\rho })=T^{*}$, the set $\left\{\rho \in N:|J_{w}^{m}({\bar{Y}}_{\rho })-T^{*}|>\varrho \right\}$ is finite for every *ϱ* > 0. Therefore, there exists $n_{0}\in N$ such that for all *ρ* > *n*_0_,

$$|J_{w}^{m}({\bar{Y}}_{\rho })-T^{*}|<\varrho .$$

Thus, $\left({\bar{Y}}_{\rho }\right)$ is $J_{w}^{m}$-convergent to $T^{*}$.

Next, since $\{\rho \in N:J_{w}^{m}({\bar{T}}_{\rho })\neq J_{w}^{m}({\bar{Y}}_{\rho })\}\subset \Omega$ and Λ(Ω) = 0, it follows that

$$\begin{array}{c} \Lambda (\{\rho \in N:J_{w}^{m}({\bar{T}}_{\rho })\neq J_{w}^{m}({\bar{Y}}_{\rho })\})=0. \end{array}$$

Conversely, for any *ϱ* > 0, we have

$$\begin{array}{c} \left\{\rho \in N:|J_{w}^{m}({\bar{T}}_{\rho })-T^{*}|>\varrho \right\}\\ \subset \{\rho \in N:J_{w}^{m}({\bar{T}}_{\rho })\neq J_{w}^{m}({\bar{Y}}_{\rho })\}\cup \{\rho \in N:|J_{w}^{m}({\bar{Y}}_{\rho })-T^{*}|>\varrho \}. \end{array}$$

Since $\left({\bar{Y}}_{\rho }\right)$ is $J_{w}^{m}$-convergent to $T^{*}$, it follows from Theorem 2.3 that this set contains only finitely many integers, say *q* = *q*(*ϱ*). Consequently,

$$\begin{array}{c} \underset{\upsilon \to \infty }{\text{lim}}\;\frac{1}{\upsilon }|\{\rho \leq \upsilon :|J_{w}^{m}({\bar{T}}_{\rho })-T^{*}|\geq \varrho \}|\\ \leq \underset{\upsilon \to \infty }{\text{lim}}\;\frac{1}{\upsilon }|\{\rho \leq \upsilon :J_{w}^{m}({\bar{T}}_{\rho })\neq J_{w}^{m}({\bar{Y}}_{\rho })\}|+\underset{\upsilon \to \infty }{\text{lim}}\;\frac{1}{\upsilon }q=0. \end{array}$$

Hence,

$$\underset{\upsilon \to \infty }{\text{lim}}\;\frac{1}{\upsilon }|\{\rho \leq \upsilon :|J_{w}^{m}({\bar{T}}_{\rho })-T^{*}|\geq \varrho \}|=0.$$

Therefore, $\left({\bar{T}}_{\rho }\right)$ is $S(J_{w}^{m})$-convergent to $T^{*}$.

**Theorem 2.6**. *If* $\left({\bar{T}}_{\rho }\right)$ *is* $S(J_{w}^{m})$–*convergent, then its* $S(J_{w}^{m})$–*limit is unique.*

*Proof*. Suppose $St(J_{w}^{m})-lim{\bar{T}}_{\rho }=T^{*}$ and $St(J_{w}^{m})-lim{\bar{T}}_{\rho }=T^{**}$. Then, for any *ϱ* > 0,

$$\underset{\upsilon \to \infty }{lim}\;\;\frac{1}{\upsilon }|\{\rho \leq \upsilon :|J_{w}^{m}({\bar{T}}_{\rho })-T^{*}|\geq \frac{\varrho }{2}\}|=0$$

and

$$\underset{\upsilon \to \infty }{lim}\;\;\frac{1}{\upsilon }|\{\rho \leq \upsilon :|J_{w}^{m}({\bar{T}}_{\rho })-T^{**}|\geq \frac{\varrho }{2}\}|=0.$$

Let us define the set Ω(*ϱ*) as

$$\Omega (\varrho )=\{\rho \leq \upsilon :|J_{w}^{m}({\bar{T}}_{\rho })-T^{*}|\geq \frac{\varrho }{2}\}\cup \{\rho \leq \upsilon :|J_{w}^{m}({\bar{T}}_{\rho })-T^{**}|\geq \frac{\varrho }{2}\}.$$

Then, Λ(Ω(*ϱ*)) = 0. So that N\Ω(ϱ)≠ϕ. Thus, for any ρ∈N\Ω(ϱ), we may write

$$|T^{*}-T^{**}|\leq |T^{*}-J_{w}^{m}({\bar{T}}_{\rho })|+|J_{w}^{m}({\bar{T}}_{\rho })-T^{**}|<\frac{\varrho }{2}+\frac{\varrho }{2}=\varrho .$$

Since *ϱ* > 0 was arbitrary, we get $|T^{*}-T^{**}|=0$, that is, $T^{*}=T^{**}$.

**Definition 2.4**. *A sequence* $\left({\bar{T}}_{\rho }\right)$ *is called Bessel statistically Cauchy (or,* $S(J_{w}^{m})$–*Cauchy) if for every ϱ* > 0, *there exists* $\rho_{0}=\rho_{0}(\varrho )\in N$ *such that*

$$\underset{\upsilon \to \infty }{lim}\;\;\frac{1}{\upsilon }|\{\rho \leq \upsilon :|J_{w}^{m}({\bar{T}}_{\rho })-J_{w}^{m}({\bar{T}}_{\rho_{0}})|\geq \varrho \}|=0.$$

**Theorem 2.7**. *A sequence* $\bar{T}=({\bar{T}}_{\rho })$ *is* $S(J_{w}^{m})$–*convergent if and only if* $\left({\bar{T}}_{\rho }\right)$ *is* $S(J_{w}^{m})$–*Cauchy*.

*Proof*. Suppose that $\bar{T}=({\bar{T}}_{\rho })$ is $S(J_{w}^{m})$–convergent to $T^{*}$. Then, for every *ϱ* > 0,

$$\underset{\upsilon \to \infty }{lim}\;\;\frac{1}{\upsilon }|\{\rho \leq \upsilon :|J_{w}^{m}({\bar{T}}_{\rho })-T^{*}|\geq \frac{\varrho }{2}\}|=0.$$

Choose a positive integer *ρ*_0_ such that $|J_{w}^{m}({\bar{T}}_{\rho_{0}})-T^{*}|\geq \varrho$. Now, for every υ∈N, we may write

$$\begin{array}{c} \frac{1}{\upsilon }|\{\rho \leq \upsilon :|J_{w}^{m}({\bar{T}}_{\rho })-J_{w}^{m}({\bar{T}}_{\rho_{0}})|\geq \frac{\varrho }{2}\}|\leq \frac{1}{\upsilon }|\{\rho \leq \upsilon :|J_{w}^{m}({\bar{T}}_{\rho })-T^{*}|\geq \frac{\varrho }{2}\}|\\ +\frac{1}{\upsilon }|\{\rho_{0}\leq \upsilon :|J_{w}^{m}({\bar{T}}_{\rho_{0}})-T^{*}|\geq \frac{\varrho }{2}\}|. \end{array}$$

By taking the limits on both sides in the above inequality, we obtain that

$$\underset{\upsilon \to \infty }{lim}\;\;\frac{1}{\upsilon }|\{\rho \leq \upsilon :|J_{w}^{m}({\bar{T}}_{\rho })-J_{w}^{m}({\bar{T}}_{\rho_{0}})|\geq \frac{\varrho }{2}\}|=0.$$

Thus, $\left({\bar{T}}_{\rho }\right)$ is $S(J_{w}^{m})$–Cauchy.

Conversely, suppose that $\left({\bar{T}}_{\rho }\right)$ is $S(J_{w}^{m})$–Cauchy. So, for every *ϱ* > 0, there exists $M_{0}\in N$ such that $\Lambda (\{\rho \in N:|J_{w}^{m}({\bar{T}}_{\rho })-J_{w}^{m}({\bar{T}}_{M_{0}})|<\varrho \})=1$. That is,

$$\Lambda (\{\rho \in N:J_{w}^{m}({\bar{T}}_{\rho })<J_{w}^{m}({\bar{T}}_{M_{0}})+\varrho \})=1$$

and

$$\Lambda (\{\rho \in N:J_{w}^{m}({\bar{T}}_{M_{0}})-\varrho <J_{w}^{m}({\bar{T}}_{\rho })\})=1.$$

Now, let us define the sets Ω_1_(*ϱ*) and Ω_2_(*ϱ*) as follows:

$$\Omega_{1}(\varrho )=\{i\in R:\Lambda (\{\rho \in N:J_{w}^{m}({\bar{T}}_{\rho })<i\})=1\}$$

and

$$\Omega_{2}(\varrho )=\{j\in R:\Lambda (\{\rho \in N:J_{w}^{m}({\bar{T}}_{\rho })>j\})=1\}.$$

Then, $\left(J_{w}^{m}({\bar{T}}_{M_{0}})+\varrho )\in \Omega_{1}(\varrho \right)$ and $\left(J_{w}^{m}({\bar{T}}_{M_{0}})-\varrho )\in \Omega_{2}(\varrho \right)$. Let *i* ∈ Ω_1_(*ϱ*) and *j* ∈ Ω_2_(*ϱ*) so that

$$\Lambda (\{\rho \in N:J_{w}^{m}({\bar{T}}_{\rho })<i\})=1\;\text{and}\;\Lambda (\{\rho \in N:J_{w}^{m}({\bar{T}}_{\rho })>j\})=1.$$

Therefore, we get $\Lambda (\{\rho \in N:j<J_{w}^{m}({\bar{T}}_{\rho })<i\})=1$. This implies *j* < *i*. So,

$$J_{w}^{m}({\bar{T}}_{M_{0}})-\varrho \leq \text{sup}\;\Omega_{2}\leq \text{inf}\;\Omega_{1}\leq J_{w}^{m}({\bar{T}}_{M_{0}})+\varrho .$$

Since *ϱ* was arbitrary, we get $\text{sup}\;\Omega_{2}\;(\varrho )=\text{inf}\;\Omega_{1}(\varrho )-T^{*}$. Now, there exist *i* ∈ Ω_1_ (*ϱ*) and *j* ∈ Ω_2_ (*ϱ*) such that $T^{*}-\varrho <j<i<T^{*}+\varrho$. From the definitions of Ω_1_ (*ϱ*) and Ω_2_ (*ϱ*), we have

$$\Lambda (\{\rho \in N:T^{*}-\varrho <J_{w}^{m}({\bar{T}}_{\rho })<T^{*}+\varrho \})=1.$$

Therefore, $\Lambda (\{\rho \in N:|J_{w}^{m}({\bar{T}}_{\rho })-T^{*}|\geq \varrho \})=0$. This means that $\left({\bar{T}}_{\rho }\right)$ is $S(J_{w}^{m})$–convergent.

## 3 Applications of Bessel statistical convergence

In this section, we extend Korovkin’s first and second theorems using Bessel statistical convergence. This approach generalizes the classical results to accommodate Bessel statistical convergence, providing a broader perspective on approximation properties. We work within the Banach space $C[u_{1},u_{2}]$ of real-valued continuous functions on [*u*_1_, *u*_2_], equipped with the supremum norm

$${\left\|g\right\|}_{C[u_{1},u_{2}]}=\underset{\xi \in R}{\;\text{sup}}\;\;|g(\xi )|$$

for g∈C(R). Now, we provide Bessel statistical analog of Korovkin first theorem, which is a new version of Korovkin first theorem of [29]. Additionally, we will demonstrate that our new theorem is significantly stronger.

**Theorem 3.1**. *Let* $\left({\bar{B}}_{\rho }\right)$ *be a sequence of positive linear operators from* $C[u_{1},u_{2}]$ *into itself. Then, for all* $g\in C[u_{1},u_{2}]$,

$$\begin{array}{c} St(J_{w}^{m})-lim{\left\|{\bar{B}}_{\rho }(g,\xi )-g(\xi )\right\|}_{C[u_{1},u_{2}]}=0 \end{array}$$ (1)

*if and only if*

$$\begin{array}{c} St(J_{w}^{m})-lim{\left\|{\bar{B}}_{\rho }(1,\xi )-1\right\|}_{C[u_{1},u_{2}]}=0, \end{array}$$ (2)

$$\begin{array}{c} St(J_{w}^{m})-lim{\left\|{\bar{B}}_{\rho }(\xi ,\xi )-\xi \right\|}_{C[u_{1},u_{2}]}=0, \end{array}$$ (3)

*and*

$$\begin{array}{c} St(J_{w}^{m})-lim{\left\|{\bar{B}}_{\rho }(\xi^{2},\xi )-\xi^{2}\right\|}_{C[u_{1},u_{2}]}=0. \end{array}$$ (4)

*Proof*. Obviously, each of the functions $g_{0}(\xi )=1$, $g_{1}(\xi )=\xi$ and $g_{2}(\xi )=\xi^{2}$ is continuous and belongs to $C[u_{1},u_{2}]$, the implication given by (1) implies (2) to (4) is clear. Now, assume that the conditions (2) to (4) hold. To show that (1) holds. Suppose that $g\in C[u_{1},u_{2}]$. Since the function g is bounded on the whole real axis so that there exists a real number *M* > 0 such that |g(ξ)|≤M for all ξ∈R. That is, for all ζ,ξ∈R,

$$\begin{array}{c} |g(\zeta )-g(\xi )|\leq 2M. \end{array}$$ (5)

Since, g is continuous, then for each *ϱ* > 0, there exists *δ* > 0 such that

$$\begin{array}{c} |g(\zeta )-g(\xi )|\leq \varrho \end{array}$$ (6)

whenever |*ξ* − *ζ*| < *δ* for all *ζ* and *ξ*. Taking *ψ*(*ζ*, *ξ*) = (*ζ* − *ξ*)^2^. If |*ξ* − *ζ*| ≥ *δ*, we obtain

$$\begin{array}{c} |g(\zeta )-g(\xi )|<\frac{2M}{\delta^{2}}\psi (\zeta ,\xi ). \end{array}$$ (7)

From the inequalities (6) and (7), we get

$$|g(\zeta )-g(\xi )|<\varrho +\frac{2M}{\delta^{2}}\psi (\zeta ,\xi ).$$

That is,

$$\begin{array}{c} -\varrho -\frac{2M}{\delta^{2}}\psi (\zeta ,\xi )\leq g(\zeta )-g(\xi )\leq \varrho +\frac{2M}{\delta^{2}}\psi (\zeta ,\xi ). \end{array}$$ (8)

By linearity and monotonicity of the linear operator ${\bar{B}}_{\rho }(g,\xi )$, the inequality (8) implies that

$$(-\varrho -\frac{2M}{\delta^{2}}\psi (\zeta ,\xi )){\bar{B}}_{\rho }(1,\xi )\leq (g(\zeta )-g(\xi )){\bar{B}}_{\rho }(1,\xi )\leq (\varrho +\frac{2M}{\delta^{2}}\psi (\zeta ,\xi )){\bar{B}}_{\rho }(1,\xi ).$$

Since *ξ* is fixed, then g(ξ) is constant. Accordingly,

$$\begin{array}{cc} -\varrho {\bar{B}}_{\rho }(1,\xi )-\frac{2M}{\delta^{2}}{\bar{B}}_{\rho }(\psi ,\xi ) & \leq {\bar{B}}_{\rho }(g,\xi )-g(\xi ){\bar{B}}_{\rho }(1,\xi )\\ & \leq \varrho {\bar{B}}_{\rho }(1,\xi )+\frac{2M}{\delta^{2}}{\bar{B}}_{\rho }(\psi ,\xi ). \end{array}$$ (9)

It is known that

$$\begin{array}{c} {\bar{B}}_{\rho }(g,\xi )-g(\xi )=({\bar{B}}_{\rho }(g,\xi )-g(\xi ){\bar{B}}_{\rho }(1,\xi ))+g(\xi )({\bar{B}}_{\rho }(1,\xi )-1). \end{array}$$ (10)

So, by using (9) and (10), we have

$$\begin{array}{c} {\bar{B}}_{\rho }(g,\xi )-g(\xi )<\varrho {\bar{B}}_{\rho }(1,\xi )+\frac{2M}{\delta^{2}}{\bar{B}}_{\rho }(\psi ,\xi )+g(\xi )({\bar{B}}_{\rho }(1,\xi )-1). \end{array}$$ (11)

By estimating ${\bar{B}}_{\rho }(\psi ,\xi )$, we may write

$$\begin{array}{cc} {\bar{B}}_{\rho }(\psi ,\xi ) & ={\bar{B}}_{\rho }(\zeta^{2}-2\zeta \xi +\xi^{2},\xi )\\ & ={\bar{B}}_{\rho }(\zeta^{2},\xi )-2\xi {\bar{B}}_{\rho }(\zeta ,\xi )+\xi^{2}{\bar{B}}_{\rho }(1,\xi )\\ & =({\bar{B}}_{\rho }(\zeta^{2},\xi )-\xi^{2})-2\xi ({\bar{B}}_{\rho }(\zeta ,\xi )-\xi )+\xi^{2}({\bar{B}}_{\rho }(1,\xi )-1). \end{array}$$ (12)

Using (11) and (12), we obtain

$$\begin{array}{cc} {\bar{B}}_{\rho }(g,\xi ) & -g(\xi )<\varrho {\bar{B}}_{\rho }(1,\xi )+\frac{2M}{\delta^{2}}[({\bar{B}}_{\rho }(\zeta^{2},\xi )-\xi^{2})-2\xi ({\bar{B}}_{\rho }(\zeta ,\xi )-\xi )\\ & \;+\xi^{2}({\bar{B}}_{\rho }(1,\xi )-1)]+g(\xi )({\bar{B}}_{\rho }(1,\xi )-1)\\ & =(\varrho +\frac{2M}{\delta^{2}}\xi^{2}+g(\xi ))({\bar{B}}_{\rho }(1,\xi )-1)+\frac{2M}{\delta^{2}}({\bar{B}}_{\rho }(\zeta^{2},\xi )-\xi^{2})\\ & \;-\frac{4M}{\delta^{2}}\xi ({\bar{B}}_{\rho }(\zeta ,\xi )-\xi )+\varrho . \end{array}$$

Thus, we have

$$\begin{array}{cc} {\left\|{\bar{B}}_{\rho }(g,\xi )-g(\xi )\right\|}_{C[u_{1},u_{2}]} & \leq \varrho +(\varrho +\frac{2M}{\delta^{2}}+M){\left\|{\bar{B}}_{\rho }(1,\xi )-1\right\|}_{C[u_{1},u_{2}]}\\ & \;+\frac{2M}{\delta^{2}}{\left\|{\bar{B}}_{\rho }(\zeta^{2},\xi )-\xi^{2}\right\|}_{C[u_{1},u_{2}]}+\frac{4M}{\delta^{2}}{\left\|{\bar{B}}_{\rho }(\zeta ,\xi )-\xi \right\|}_{C[u_{1},u_{2}]}\\ & \leq \varrho +K({\left\|{\bar{B}}_{\rho }(1,\xi )-1\right\|}_{C[u_{1},u_{2}]}+{\left\|{\bar{B}}_{\rho }(\zeta^{2},\xi )-\xi^{2}\right\|}_{C[u_{1},u_{2}]}\\ & \;+{\left\|{\bar{B}}_{\rho }(\zeta ,\xi )-\xi \right\|}_{C[u_{1},u_{2}]}), \end{array}$$

when $K=\text{max}\;\{\varrho +\frac{2M}{\delta^{2}}+M,\frac{2M}{\delta^{2}},\frac{4M}{\delta^{2}}\}$. Hence,

$$\begin{array}{c} {\left\|\sum_{u=0}^{m}\frac{{\left(-1\right)}^{u}}{u!\Gamma (u+w+1)}{\left(\frac{{\bar{B}}_{\rho +u}(g,\xi )}{2}\right)}^{2u+w}-g(\xi )\right\|}_{C[u_{1},u_{2}]}\\ \leq \varrho +K({\left\|\sum_{u=0}^{m}\frac{{\left(-1\right)}^{u}}{u!\Gamma (u+w+1)}{\left(\frac{{\bar{B}}_{\rho +u}(1,\xi )}{2}\right)}^{2u+w}-1\right\|}_{C[u_{1},u_{2}]}\\ \;+{\left\|\sum_{u=0}^{m}\frac{{\left(-1\right)}^{u}}{u!\Gamma (u+w+1)}{\left(\frac{{\bar{B}}_{\rho +u}(\zeta^{2},\xi )}{2}\right)}^{2u+w}-\xi^{2}\right\|}_{C[u_{1},u_{2}]}\\ \;+{\left\|\sum_{u=0}^{m}\frac{{\left(-1\right)}^{u}}{u!\Gamma (u+w+1)}{\left(\frac{{\bar{B}}_{\rho +u}(\zeta ,\xi )}{2}\right)}^{2u+w}-\xi \right\|}_{C[u_{1},u_{2}]}). \end{array}$$

Or,

$$\begin{array}{cc} & {\left\|J_{w}^{m}({\bar{B}}_{\rho }(g,\xi ))-g(\xi )\right\|}_{C[u_{1},u_{2}]}\\ & \leq \varrho +K({\left\|J_{w}^{m}({\bar{B}}_{\rho }(1,\xi ))-1\right\|}_{C[u_{1},u_{2}]}+{\left\|J_{w}^{m}({\bar{B}}_{\rho }(\zeta^{2},\xi ))-\xi^{2}\right\|}_{C[u_{1},u_{2}]}\\ & \;+{\left\|J_{w}^{m}({\bar{B}}_{\rho }(\zeta ,\xi ))-\xi \right\|}_{C[u_{1},u_{2}]}). \end{array}$$ (13)

For any *ϱ*′ > 0, we may choose *ϱ* > 0 such that *ϱ* < *ϱ*′. From (13), we have

$$\begin{array}{cc} & \left\{\rho \in N:{\left\|J_{w}^{m}({\bar{B}}_{\rho }(g,\xi ))-g(\xi )\right\|}_{C[u_{1},u_{2}]}\geq \varrho^{\prime }\right\}\\ & \subset \{\rho \in N:{\left\|J_{w}^{m}({\bar{B}}_{\rho }(1,\xi ))-1\right\|}_{C[u_{1},u_{2}]}+{\left\|J_{w}^{m}({\bar{B}}_{\rho }(\zeta ,\xi ))-\xi \right\|}_{C[u_{1},u_{2}]}\\ & \;+{\left\|J_{w}^{m}({\bar{B}}_{\rho }(\zeta^{2},\xi ))-\xi^{2}\right\|}_{C[u_{1},u_{2}]}\geq \frac{\varrho^{\prime }-\varrho }{K}\}. \end{array}$$ (14)

Now, let us take

$$\begin{array}{cc} \Omega & =\{\rho \in N:{\left\|J_{w}^{m}({\bar{B}}_{\rho }(1,\xi ))-1\right\|}_{C[u_{1},u_{2}]}+{\left\|J_{w}^{m}({\bar{B}}_{\rho }(\zeta ,\xi ))-\xi \right\|}_{C[u_{1},u_{2}]}\\ & +{\left\|J_{w}^{m}({\bar{B}}_{\rho }(\zeta^{2},\xi ))-\xi^{2}\right\|}_{C[u_{1},u_{2}]}\geq \frac{\varrho^{\prime }-\varrho }{K}\}, \end{array}$$

$$\Omega_{1}=\{\rho \in N:{\left\|J_{w}^{m}({\bar{B}}_{\rho }(1,\xi ))-1\right\|}_{C[u_{1},u_{2}]}\geq \frac{\varrho^{\prime }-\varrho }{3K}\},$$

$$\Omega_{2}=\{\rho \in N:{\left\|J_{w}^{m}({\bar{B}}_{\rho }(\zeta ,\xi ))-\xi \right\|}_{C[u_{1},u_{2}]}\geq \frac{\varrho^{\prime }-\varrho }{3K}\},$$

and

$$\Omega_{3}=\{\rho \in N:{\left\|J_{w}^{m}({\bar{B}}_{\rho }(\zeta^{2},\xi ))-\xi^{2}\right\|}_{C[u_{1},u_{2}]}\geq \frac{\varrho^{\prime }-\varrho }{3K}\}.$$

Clearly, we have Ω ⊂ Ω_1_ ∪ Ω_2_ ∪ Ω_3_. So that (14) implies

$$\begin{array}{c} \frac{1}{\upsilon }|\{\rho \in N:{\left\|J_{w}^{m}({\bar{B}}_{\rho }(g,\xi ))-g(\xi )\right\|}_{C[u_{1},u_{2}]}\geq \varrho^{\prime }\}|\\ \leq \frac{1}{\upsilon }|\{\rho \in N:{\left\|J_{w}^{m}({\bar{B}}_{\rho }(1,\xi ))-1\right\|}_{C[u_{1},u_{2}]}\geq \frac{\varrho^{\prime }-\varrho }{3K}\}|\\ \;+\frac{1}{\upsilon }|\{\rho \in N:{\left\|J_{w}^{m}({\bar{B}}_{\rho }(\zeta ,\xi ))-\xi \right\|}_{C[u_{1},u_{2}]}\geq \frac{\varrho^{\prime }-\varrho }{3K}\}|\\ \;+\frac{1}{\upsilon }|\{\rho \in N:{\left\|J_{w}^{m}({\bar{B}}_{\rho }(\zeta^{2},\xi ))-\xi^{2}\right\|}_{C[u_{1},u_{2}]}\geq \frac{\varrho^{\prime }-\varrho }{3K}\}|. \end{array}$$

By taking the limits as *υ* → ∞ and using the above assumption for the implications (2) to (4), we obtain that

$$\underset{\upsilon \to \infty }{lim}\;\;\frac{1}{\upsilon }|\{\rho \leq \upsilon :{\left\|J_{w}^{m}({\bar{B}}_{\rho }(g,\xi ))-g(\xi )\right\|}_{C[u_{1},u_{2}]}\geq \varrho^{\prime }\}|=0.$$

Therefore,

$$\begin{array}{c} St(J_{w}^{m})-lim{\left\|{\bar{B}}_{\rho }(g,\xi )-g(\xi )\right\|}_{C[u_{1},u_{2}]}=0. \end{array}$$

This completes the proof.

As illustrated in the forthcoming example, it is feasible to construct a sequence of positive linear operators that meets the criteria of Theorem 3.1 but fails to satisfy the requirements of the classical Korovkin approximation theorem, as detailed in [29]. This approach highlights that our result offers a broader scope than the classical results.

**Example 3.1**. *Consider the sequence of Bernstein operators*

$$B_{\upsilon }(g,\xi )=\sum_{\rho =0}^{\upsilon }g(\frac{\rho }{\upsilon })(\begin{array}{c} \upsilon \\ \rho \end{array})\xi^{\rho }{\left(1-\xi \right)}^{\upsilon -\rho },\;\xi \in [0,1].$$

*Define the sequence* $\left({\bar{T}}_{\upsilon }\right)$ *such that* $St\left(J_{w}^{m}\right)-lim{\bar{T}}_{\upsilon }=0$ *and* $J_{w}^{m}-lim{\bar{T}}_{\upsilon }\neq 0$ *(this condition is permissible as indicated by Theorem 2.3), and define the sequence of linear operators* ${\bar{W}}_{\upsilon }:C[0,1]\to C[0,1]$ *by*

$${\bar{W}}_{\upsilon }(g,\xi )=(1+J_{w}^{m}({\bar{T}}_{\upsilon }))B_{\upsilon }(g,\xi ).$$

*It is established (refer to* [29]*) that*

$$B_{\upsilon }(1,\xi )=1,\;B_{\upsilon }(\xi ,\xi )=\xi ,B_{\upsilon }(\xi^{2},\xi )=\xi^{2}+\frac{\xi -\xi^{2}}{\upsilon }.$$

*That is,*

$${\bar{W}}_{\upsilon }(1,\xi )=(1+J_{w}^{m}({\bar{T}}_{\upsilon }))B_{\upsilon }(1,\xi ),$$

$${\bar{W}}_{\upsilon }(\xi ,\xi )=(1+J_{w}^{m}({\bar{T}}_{\upsilon }))B_{\upsilon }(\xi ,\xi ),$$

*and*

$${\bar{W}}_{\upsilon }(\xi^{2},\xi )=(1+J_{w}^{m}({\bar{T}}_{\upsilon }))B_{\upsilon }(\xi^{2},\xi ).$$

*This implies that*

$$St(J_{w}^{m})-lim{\left\|{\bar{W}}_{\upsilon }(1,\xi )-1\right\|}_{C[u_{1},u_{2}]}=0,$$

$$St(J_{w}^{m})-lim{\left\|{\bar{W}}_{\upsilon }(\xi ,\xi )-\xi \right\|}_{C[u_{1},u_{2}]}=0,$$

*and*

$$St(J_{w}^{m})-lim{\left\|{\bar{W}}_{\upsilon }(\xi^{2},\xi )-\xi^{2}\right\|}_{C[u_{1},u_{2}]}=0.$$

*Therefore, according to Theorem 3.1, we get*

$$St(J_{w}^{m})-lim{\left\|{\bar{W}}_{\upsilon }(g,\xi )-g(\xi )\right\|}_{C[u_{1},u_{2}]}=0.$$

*On the other hand, we have*

$$lim\left\|{\bar{W}}_{\upsilon }(1,\xi )-1\right\|=lim\left\|1+J_{w}^{m}({\bar{T}}_{\upsilon })-1\right\|=lim\left\|J_{w}^{m}({\bar{T}}_{\upsilon })\right\|=lim|J_{w}^{m}({\bar{T}}_{\upsilon })|\neq 0.$$

*This implies that the sequence* $\left({\bar{W}}_{\upsilon }\right)$ *does not fulfill the conditions of the classical Korovkin theorem*.

We now present a Bessel statistical version of Korovkin’s second theorem. Let $C_{2\pi }(R)$ represent the space of all 2*π*-periodic functions g∈C(R), which forms a Banach space with the norm given by ${\left\|g\right\|}_{2\pi }=\underset{\xi \in R}{\text{sup}}\;\;|g(\xi )|$, where $g\in C_{2\pi }(R)$.

**Theorem 3.2**. *Consider a sequence of positive linear operators* $\left({\bar{B}}_{\rho }\right)$ *such that* ${\bar{B}}_{\rho }:C_{2\pi }(R)\to C_{2\pi }(R)$. *Then, for every* $g\in C_{2\pi }(R)$,

$$\begin{array}{c} St(J_{w}^{m})-lim{\left\|{\bar{B}}_{\rho }(g,\xi )-g(\xi )\right\|}_{2\pi }=0 \end{array}$$ (15)

*if and only if*

$$\begin{array}{c} St(J_{w}^{m})-lim{\left\|{\bar{B}}_{\rho }(1,\xi )-1\right\|}_{2\pi }=0, \end{array}$$ (16)

$$\begin{array}{c} St(J_{w}^{m})-lim{\left\|{\bar{B}}_{\rho }(\text{cos}\;\zeta ,\xi )-\text{cos}\;\xi \right\|}_{2\pi }=0, \end{array}$$ (17)

*and*

$$\begin{array}{c} St(J_{w}^{m})-lim{\left\|{\bar{B}}_{\rho }(\text{sin}\;\zeta ,\xi )-\text{sin}\;\xi \right\|}_{2\pi }=0. \end{array}$$ (18)

*Proof*. Conditions (16) to (18) hold immediately from (15) since the functions $1,\text{sin}\;\xi ,\text{cos}\;\xi \in C_{2\pi }(R)$.

Conversely, assume that the conditions (16) to (18) hold. We shall prove that (15) holds. For this, let us take $g\in C_{2\pi }(R)$. To prove

$$St(J_{w}^{m})-lim{\left\|{\bar{B}}_{\rho }(g,\xi )-g(\xi )\right\|}_{2\pi }=0.$$

Let’s consider *U* = (*ξ* − *δ*, 2*π* + *ξ* − *δ*] of the length 2*π* of R and *ξ* ∈ *U* is fixed. Since the function g is bounded on R, for all ζ∈R,

$$\begin{array}{c} |g(\zeta )-g(\xi )|<2{\left\|g\right\|}_{2\pi }. \end{array}$$ (19)

Also, since g is continuous at *ξ*, for each *ϱ* > 0, there exists *δ* > 0 such that

$$\begin{array}{c} |g(\zeta )-g(\xi )|\leq \varrho . \end{array}$$ (20)

whenever ζ∈R and |*ξ* − *ζ*| < *δ*. Now, take $\psi (\zeta )={\text{sin}}^{2}\;\frac{\left(\zeta -\xi \right)}{2}$. By using (19) and (20), for all *ζ* ∈ *U*, we may write

$$\begin{array}{c} |g(\zeta )-g(\xi )|<\varrho +\frac{2{\left\|g\right\|}_{2\pi }}{{\text{sin}}^{2}\;\frac{\delta }{2}}\psi (\zeta ). \end{array}$$ (21)

That is, for all *ζ* ∈ *U*,

$$\begin{array}{c} -\varrho -\frac{2{\left\|g\right\|}_{2\pi }}{{\text{sin}}^{2}\frac{\delta }{2}}\psi (\zeta )<g(\zeta )-g(\xi )<\varrho +\frac{2{\left\|g\right\|}_{2\pi }}{{\text{sin}}^{2}\;\frac{\delta }{2}}\psi (\zeta ). \end{array}$$ (22)

By linearity and positivity of ${\bar{B}}_{\rho }(g,\xi )$, the inequality (22) can be written as

$$\begin{array}{cc} -\varrho {\bar{B}}_{\rho }(1,\xi )-\frac{2{\left\|g\right\|}_{2\pi }}{{\text{sin}}^{2}\;\frac{\delta }{2}}{\bar{B}}_{\rho }(\psi ,\xi ) & <{\bar{B}}_{\rho }(g,\xi )-{\bar{B}}_{\rho }(g(\xi ),\xi )\\ & <\varrho {\bar{B}}_{\rho }(1,\xi )+\frac{2{\left\|g\right\|}_{2\pi }}{{\text{sin}}^{2}\;\frac{\delta }{2}}{\bar{B}}_{\rho }(\psi ,\xi ). \end{array}$$ (23)

Since *ξ* is fixed, so that g(ξ) is a constant number. That is, (23) implies

$$\begin{array}{cc} -\varrho {\bar{B}}_{\rho }(1,\xi )-\frac{2{\left\|g\right\|}_{2\pi }}{{\text{sin}}^{2}\frac{\delta }{2}}{\bar{B}}_{\rho }(\psi ,\xi ) & <{\bar{B}}_{\rho }(g,\xi )-{\bar{B}}_{\rho }(1,\xi )g(\xi )\\ & <\varrho {\bar{B}}_{\rho }(1,\xi )+\frac{2{\left\|g\right\|}_{2\pi }}{{\text{sin}}^{2}\frac{\delta }{2}}{\bar{B}}_{\rho }(\psi ,\xi ). \end{array}$$ (24)

On the other hand,

$$\begin{array}{c} {\bar{B}}_{\rho }(g,\xi )-g(\xi )={\bar{B}}_{\rho }(g,\xi )-g(\xi ){\bar{B}}_{\rho }(1,\xi )+g(\xi )({\bar{B}}_{\rho }(1,\xi )-1). \end{array}$$ (25)

From the inequality (24) and the equality (25), we get

$$\begin{array}{c} {\bar{B}}_{\rho }(g,\xi )-g(\xi )<\varrho {\bar{B}}_{\rho }(1,\xi )+\frac{2{\left\|g\right\|}_{2\pi }}{{\text{sin}}^{2}\frac{\delta }{2}}{\bar{B}}_{\rho }(\psi ,\xi )+g(\xi )({\bar{B}}_{\rho }(1,\xi )-1). \end{array}$$ (26)

Now,

$$\begin{array}{cc} {\bar{B}}_{\rho }(\psi ,\xi ) & ={\bar{B}}_{\rho }({\text{sin}}^{2}\frac{\left(\zeta -\xi \right)}{2},\xi )\\ & ={\bar{B}}_{\rho }(\frac{1}{2}(1-\text{cos}\;\zeta \;\text{cos}\;\xi -\text{sin}\;\zeta \;\text{sin}\;\xi ),\xi )\\ & =\frac{1}{2}[{\bar{B}}_{\rho }(1,\xi )-1-\text{cos}\;\xi ({\bar{B}}_{\rho }(\text{cos}\;\zeta ,\xi )-\text{cos}\;\xi )-\text{sin}\;\xi ({\bar{B}}_{\rho }(\text{sin}\;\zeta ,\xi )-\text{sin}\;\xi )]. \end{array}$$

Using ${\bar{B}}_{\rho }(\psi ,\xi )$ in (26), we may write

$$\begin{array}{cc} {\bar{B}}_{\rho }(g,\xi )-g(\xi )< & \varrho {\bar{B}}_{\rho }(1,\xi )+\frac{{\left\|g\right\|}_{2\pi }}{{\text{sin}}^{2}\frac{\delta }{2}}[{\bar{B}}_{\rho }(1,\xi )-1-\text{cos}\;\xi ({\bar{B}}_{\rho }(\text{cos}\;\zeta ,\xi )-\text{cos}\;\xi )\\ & \;-\text{sin}\;\xi ({\bar{B}}_{\rho }(\text{sin}\;\zeta ,\xi )-\text{sin}\;\xi )]+g(\xi )({\bar{B}}_{\rho }(1,\xi )-1)\\ & =(\varrho +\frac{{\left\|g\right\|}_{2\pi }}{{\text{sin}}^{2}\frac{\delta }{2}}+g(\xi ))({\bar{B}}_{\rho }(1,\xi )-1)-\frac{{\left\|g\right\|}_{2\pi }}{{\text{sin}}^{2}\frac{\delta }{2}}\text{cos}\;\xi [{\bar{B}}_{\rho }(\text{cos}\;\zeta ,\xi )-\text{cos}\;\xi ]\\ & \;-\frac{{\left\|g\right\|}_{2\pi }}{{\text{sin}}^{2}\frac{\delta }{2}}\text{sin}\;\xi [{\bar{B}}_{\rho }(\text{sin}\;\zeta ,\xi )-\text{sin}\;\xi ]+\varrho . \end{array}$$

So, from the above inequality, we have

$$\begin{array}{cc} {\left\|{\bar{B}}_{\rho }(g,\xi )-g(\xi )\right\|}_{2\pi } & \leq (\varrho +\frac{{\left\|g\right\|}_{2\pi }}{{\text{sin}}^{2}\frac{\delta }{2}}+{\left\|g\right\|}_{2\pi })\|{\bar{B}}_{\rho }(1,\xi )-1\|+\frac{{\left\|g\right\|}_{2\pi }}{{\text{sin}}^{2}\frac{\delta }{2}}{\left\|{\bar{B}}_{\rho }(\text{cos}\;\zeta ,\xi )-\text{cos}\;\xi \right\|}_{2\pi }\\ & \;+\frac{{\left\|g\right\|}_{2\pi }}{{\text{sin}}^{2}\frac{\delta }{2}}{\left\|{\bar{B}}_{\rho }(\text{sin}\;\zeta ,\xi )-\text{sin}\;\xi \right\|}_{2\pi }+\varrho \\ & \leq K({\left\|{\bar{B}}_{\rho }(1,\xi )-1\right\|}_{2\pi }+{\left\|{\bar{B}}_{\rho }(\text{cos}\;\zeta ,\xi )-\text{cos}\;\xi \right\|}_{2\pi }\\ & \;+{\left\|{\bar{B}}_{\rho }(\text{sin}\;\zeta ,\xi )-\text{sin}\;\xi \right\|}_{2\pi })+\varrho , \end{array}$$

where $K=\text{max}\;\{\varrho +\frac{{\left\|g\right\|}_{2\pi }}{{\text{sin}}^{2}\frac{\delta }{2}}+{\left\|g\right\|}_{2\pi },\frac{{\left\|g\right\|}_{2\pi }}{{\text{sin}}^{2}\frac{\delta }{2}}\}$. Hence,

$$\begin{array}{cc} & {\left\|\sum_{u=0}^{m}\frac{{\left(-1\right)}^{u}}{u!\Gamma (u+w+1)}{\left(\frac{{\bar{B}}_{\rho +u}(g,\xi )}{2}\right)}^{2u+w}-g(\xi )\right\|}_{2\pi }\\ & \leq K({\left\|\sum_{u=0}^{m}\frac{{\left(-1\right)}^{u}}{u!\Gamma (u+w+1)}{\left(\frac{{\bar{B}}_{\rho +u}(1,\xi )}{2}\right)}^{2u+w}-1\right\|}_{2\pi }\\ & \;+{\left\|\sum_{u=0}^{m}\frac{{\left(-1\right)}^{u}}{u!\Gamma (u+w+1)}{\left(\frac{{\bar{B}}_{\rho +u}(\text{cos}\;\zeta ,\xi )}{2}\right)}^{2u+w}-\text{cos}\;\xi \right\|}_{2\pi }\\ & \;+{\left\|\sum_{u=0}^{m}\frac{{\left(-1\right)}^{u}}{u!\Gamma (u+w+1)}{\left(\frac{{\bar{B}}_{\rho +u}(\text{sin}\;\zeta ,\xi )}{2}\right)}^{2u+w}-\text{sin}\;\xi \right\|}_{2\pi })+\varrho . \end{array}$$ (27)

This means,

$$\begin{array}{c} {\left\|J_{w}^{m}({\bar{B}}_{\rho }(g,\xi ))-g(\xi )\right\|}_{2\pi }\\ \leq K({\left\|J_{w}^{m}({\bar{B}}_{\rho }(1,\xi ))-1\right\|}_{2\pi }+{\left\|J_{w}^{m}({\bar{B}}_{\rho }(\text{cos}\;\zeta ,\xi ))-\text{cos}\;\xi \right\|}_{2\pi }\\ +{\left\|J_{w}^{m}({\bar{B}}_{\rho }(\text{sin}\;\zeta ,\xi ))-\text{sin}\;\xi \right\|}_{2\pi })+\varrho . \end{array}$$

For any *ϱ*′ > 0, choose *ϱ* > 0 such that *ϱ* < *ϱ*′. Now, from the inequality (27), we get

$$\begin{array}{c} |\{\rho \leq \upsilon :{\left\|J_{w}^{m}({\bar{B}}_{\rho }(g,\xi ))-g(\xi )\right\|}_{2\pi }\\ \geq \varrho^{\prime }\}|\leq |\{\rho \leq \upsilon :{\left\|J_{w}^{m}({\bar{B}}_{\rho }(1,\xi ))-1\right\|}_{2\pi }+{\left\|J_{w}^{m}({\bar{B}}_{\rho }(\text{cos}\;\zeta ,\xi ))-\text{cos}\;\xi \right\|}_{2\pi }\\ \;+{\left\|J_{w}^{m}({\bar{B}}_{\rho }(\text{sin}\;\zeta ,\xi ))-\text{sin}\;\xi \right\|}_{2\pi }\geq \frac{\varrho^{\prime }-\varrho }{K}\}|. \end{array}$$

Define the following sets

$$\Omega =\{\rho \in N:{\left\|J_{w}^{m}({\bar{B}}_{\rho }(g,\xi ))-g(\xi )\right\|}_{2\pi }\geq \varrho^{\prime }\},$$

$$\Omega_{1}=\{\rho \in N:{\left\|J_{w}^{m}({\bar{B}}_{\rho }(1,\xi ))-1\right\|}_{2\pi }\geq \frac{\varrho^{\prime }-\varrho }{3K}\},$$

$$\Omega_{2}=\{\rho \in N:{\left\|J_{w}^{m}({\bar{B}}_{\rho }(\text{cos}\;\zeta ,\xi ))-\text{cos}\;\xi \right\|}_{2\pi }\geq \frac{\varrho^{\prime }-\varrho }{3K}\},$$

$$\Omega_{3}=\{\rho \in N:{\left\|J_{w}^{m}({\bar{B}}_{\rho }(\text{sin}\;\zeta ,\xi ))-\text{sin}\;\xi \right\|}_{2\pi }\geq \frac{\varrho^{\prime }-\varrho }{3K}\}.$$

It is clear that Ω ⊂ Ω_1_ ∪ Ω_2_ ∪ Ω_3_. Thus, we may write

$$\begin{array}{cc} & \frac{1}{\upsilon }|\{\rho \leq \upsilon :{\left\|J_{w}^{m}({\bar{B}}_{\rho }(g,\xi ))-g(\xi )\right\|}_{2\pi }\geq \varrho^{\prime }\}|\\ & \leq \frac{1}{\upsilon }|\{\rho \leq \upsilon :{\left\|J_{w}^{m}({\bar{B}}_{\rho }(1,\xi ))-1\right\|}_{2\pi }\geq \frac{\varrho^{\prime }-\varrho }{3K}\}|\\ & \;+\frac{1}{\upsilon }|\{\rho \leq \upsilon :{\left\|J_{w}^{m}({\bar{B}}_{\rho }(\text{cos}\;\zeta ,\xi ))-\text{cos}\;\xi \right\|}_{2\pi }\geq \frac{\varrho^{\prime }-\varrho }{3K}\}|\\ & \;+\frac{1}{\upsilon }|\{\rho \leq \upsilon :{\left\|J_{w}^{m}({\bar{B}}_{\rho }(\text{sin}\;\zeta ,\xi ))-\text{sin}\;\xi \right\|}_{2\pi }\geq \frac{\varrho^{\prime }-\varrho }{3K}\}|. \end{array}$$ (28)

Given that (16) through (18) are satisfied, by allowing *υ* → ∞ and applying this limit to both sides of the inequality in (28), we derive

$$St(J_{w}^{m})-lim{\left\|{\bar{B}}_{\rho }(g,\xi )-g(\xi )\right\|}_{2\pi }=0.$$

The subsequent example illustrates the existence of a sequence of positive linear operators that meet the criteria of Theorem 3.2, yet fail to satisfy the requirements of the classical second Korovkin theorem as presented in [29]. This indicates that our result is significantly more robust.

**Example 3.2**. *For* υ∈N, $S_{\upsilon }(g)$ *denote the υ*^*th*^–*partial sum of the Fourier series of a function* g, *i.e*.,

$$S_{\upsilon }(g,\xi )=\frac{1}{2}a_{0}(g)+\sum_{\rho =0}^{\upsilon }a_{\rho }(g)\;\text{cos}\;\rho \xi +b_{\rho }(g)\;\text{sin}\;\rho \xi .$$

*Consider the sequence of linear operators* ${\bar{D}}_{\upsilon }:C_{2\pi }(R)\to C_{2\pi }(R)$ *defined by*

$${\bar{D}}_{\upsilon }(g,\xi )=(1+{\bar{T}}_{\upsilon })F_{\upsilon }(g,\xi ),$$

*where* $\left({\bar{T}}_{\upsilon }\right)$ *is the sequence of scalars that is* $S(J_{w}^{m})$–*convergent to zero but not* $J_{w}^{m}$–*convergent to zero*, ${\left(F_{\upsilon }\right)}_{\rho =1}^{\infty }$ *is the sequence of Fejér convolution operators defined by*

$$F_{\upsilon }(g,\xi )=\frac{1}{2\pi }\int_{-\pi }^{\pi }g(\zeta )\varphi_{\upsilon }(\xi -\zeta )dt,$$

*and* ${\left(\varphi_{\upsilon }\right)}_{\rho =1}^{\infty }$ *is a positive kernal which is called Fejér kernal defined by*

$$\varphi_{\upsilon }(\xi )=\{\begin{array}{cc} \upsilon +1, & if\;\xi \;is\;a\;multiple\;of\;2\pi ,\\ \frac{{\text{sin}}^{2}(\frac{\left(\upsilon +1)(\xi -\zeta \right)}{2})}{\left(\upsilon +1\right){\text{sin}}^{2}(\frac{\xi -\zeta }{2})}, & if\;\xi \;is\;not\;multiple\;of\;2\pi . \end{array}$$

*Now, we have* ${\bar{D}}_{\upsilon }(1,\xi )=1$, ${\bar{D}}_{\upsilon }(\text{sin}\;\zeta ,\xi )=\frac{\upsilon }{\upsilon +1}\text{sin}\;\xi$ *and* ${\bar{D}}_{\upsilon }(\text{cos}\;\zeta ,\xi )=\frac{\upsilon }{\upsilon +1}\text{cos}\;\xi$. *That is, the sequence* $\left({\bar{D}}_{\upsilon }\right)$ *satisfies the conditions* (16) *to* (18). *So that*

$$St(J_{w}^{m})-lim{\left\|{\bar{D}}_{\upsilon }(g,\xi )-g(\xi )\right\|}_{2\pi }=0.$$

*On the other hand, we have*

$${\bar{D}}_{\upsilon }(1,\xi )=(\bar{T}+\infty_{\upsilon })F_{\upsilon }(1,\xi )=(\bar{T}+\infty_{\upsilon }),$$

*This implies that*

$$lim{\left\|{\bar{D}}_{\upsilon }(1,\xi )-1\right\|}_{2\pi }=lim\left\|{\bar{T}}_{\upsilon }+1-1\right\|=lim\left\|{\bar{T}}_{\upsilon }\right\|=lim|{\bar{T}}_{\upsilon }|\neq 0.$$

*As a result, it follows that the sequence* $\left({\bar{D}}_{\upsilon }\right)$ *does not fulfill the conditions of the classical Korovkin second theorem of* [29].

**Definition 3.1**. *Let* 0 < *μ* < 1. *Then, a sequence* $\left({\bar{T}}_{\rho }\right)$ *is called Bessel statistically convergent with degree μ* (*or briefly*, $S(J_{w}^{m(1-\mu )})$–*convergent) to a number* $T^{*}$ *if for every ϱ* > 0,

$$\underset{\upsilon \to \infty }{lim}\;\;\frac{1}{\upsilon^{1-\mu }}|\{\rho \leq \upsilon :|J_{w}^{m}({\bar{T}}_{\rho })-T^{*}|\geq \varrho \}|=0.$$

*In this case, we write* $St(J_{w}^{m(1-\mu )})-lim{\bar{T}}_{\rho }=T^{*}$. *Throughout the study, the class of all* $S(J_{w}^{m(1-\mu )})$–*convergent sequences is denoted by* $S[J_{w}^{m(1-\mu )}]$.

**Theorem 3.3**. *Let μ*_1_, *μ*_2_ ∈ (0, 1). *If* $\left({\bar{T}}_{\rho }\right)$ *and* $\left({\bar{Y}}_{\rho }\right)$ *are two sequences such that* $St(J_{w}^{m(1-\mu_{1})})-lim{\bar{T}}_{\rho }=T^{*}$ *and* $St(J_{w}^{m(1-\mu_{2})})-lim{\bar{Y}}_{\rho }=Y^{*}$. *Then*:

1.
$St(J_{w}^{m(1-\mu_{1})})-lim({\bar{T}}_{\rho }+{\bar{Y}}_{\rho })=T^{*}+Y^{*}$ , *where* *μ* = min {*μ*_1_, *μ*_2_}.
2.
$St(J_{w}^{m(1-\mu_{1})})-limc{\bar{T}}_{\rho }=cT^{*}$ *for any number c*.

*Proof*. Straightforward.

**Theorem 3.4**. *Let μ* ∈ (0, 1). *If a sequence* $\left({\bar{T}}_{\rho }\right)$ *is* $S(J_{w}^{m(1-\mu )})$–*convergent, then it is* $S(J_{w}^{m})$–*convergent*.

*Proof*. Straightforward.

**Theorem 3.5**. *Let* *μ*_1_, *μ*_2_, *μ*_3_ ∈ (0, 1), *and let* $\left({\bar{B}}_{\rho }\right)$ *be a sequence of positive linear operators from* $C[u_{1},u_{2}]$ *into itself such that*

$$\begin{array}{c} St(J_{w}^{m(1-\mu_{1})})-lim{\left\|{\bar{B}}_{\rho }(1,\xi )-1\right\|}_{C[u_{1},u_{2}]}=0, \end{array}$$ (29)

$$\begin{array}{c} St(J_{w}^{m(1-\mu_{2})})-lim{\left\|{\bar{B}}_{\rho }(\xi ,\xi )-\xi \right\|}_{C[u_{1},u_{2}]}=0, \end{array}$$ (30)

*and*

$$\begin{array}{c} St(J_{w}^{m(1-\mu_{3})})-lim{\left\|{\bar{B}}_{\rho }(\xi^{2},\xi )-\xi^{2}\right\|}_{C[u_{1},u_{2}]}=0. \end{array}$$ (31)

*Then, for all* $g\in C[u_{1},u_{2}]$,

$$\begin{array}{c} St(J_{w}^{m(1-\mu )})-lim{\left\|{\bar{B}}_{\rho }(g,\xi )-g(\xi )\right\|}_{C[u_{1},u_{2}]}=0, \end{array}$$

*where* *μ* = min {*μ*_1_, *μ*_2_, *μ*_3_}.

*Proof*. By using the same techniques of Theorem 3.1, for each υ∈N, we get

$$\begin{array}{c} \frac{1}{\upsilon^{1-\mu }}|\{\rho \leq \upsilon :{\left\|J_{w}^{m}(\bar{B}(g,\xi ))-g(\xi )\right\|}_{C[u_{1},u_{2}]}\geq \varrho^{\prime }\}|\\ \leq \frac{1}{\upsilon^{1-\mu }}|\{\rho \leq \upsilon :{\left\|J_{w}^{m}(\bar{B}(1,\xi ))-1\right\|}_{C[u_{1},u_{2}]}\geq \frac{\varrho^{\prime }-\varrho }{3K}\}|\\ +\frac{1}{\upsilon^{1-\mu }}|\{\rho \leq \upsilon :{\left\|J_{w}^{m}(\bar{B}(\zeta ,\xi ))-\xi \right\|}_{C[u_{1},u_{2}]}\geq \frac{\varrho^{\prime }-\varrho }{3K}\}|\\ +\frac{1}{\upsilon^{1-\mu }}|\{\rho \leq \upsilon :{\left\|J_{w}^{m}(\bar{B}(\zeta^{2},\xi ))-\xi^{2}\right\|}_{C[u_{1},u_{2}]}\geq \frac{\varrho^{\prime }-\varrho }{3K}\}|. \end{array}$$

This implies that

$$\begin{array}{c} \frac{1}{\upsilon^{1-\mu }}|\{\rho \leq \upsilon :{\left\|J_{w}^{m}(\bar{B}(g,\xi ))-g(\xi )\right\|}_{C[u_{1},u_{2}]}\geq \varrho^{\prime }\}|\\ \leq \frac{1}{\upsilon^{1-\mu_{1}}}|\{\rho \leq \upsilon :{\left\|J_{w}^{m}(\bar{B}(1,\xi ))-1\right\|}_{C[u_{1},u_{2}]}\geq \frac{\varrho^{\prime }-\varrho }{3K}\}|(\frac{\upsilon^{1-\mu_{1}}}{\upsilon^{1-\mu }})\\ +\frac{1}{\upsilon^{1-\mu_{2}}}|\{\rho \leq \upsilon :{\left\|J_{w}^{m}(\bar{B}(\zeta ,\xi ))-\xi \right\|}_{C[u_{1},u_{2}]}\geq \frac{\varrho^{\prime }-\varrho }{3K}\}|(\frac{\upsilon^{1-\mu_{2}}}{\upsilon^{1-\mu }})\\ +\frac{1}{\upsilon^{1-\mu_{3}}}|\{\rho \leq \upsilon :{\left\|J_{w}^{m}(\bar{B}(\zeta^{2},\xi ))-\xi^{2}\right\|}_{C[u_{1},u_{2}]}\geq \frac{\varrho^{\prime }-\varrho }{3K}\}|(\frac{\upsilon^{1-\mu_{3}}}{\upsilon^{1-\mu }}). \end{array}$$

By using the conditions (29) to (31), we obtain

$$St(J_{w}^{m(1-\mu )})-lim{\left\|{\bar{B}}_{\rho }(g,\xi )-g(\xi )\right\|}_{C[u_{1},u_{2}]}=0.$$

## 4 Conclusions and suggestions for further studies

In this research paper, we have introduced the concepts of Bessel convergence, Bessel boundedness, Bessel statistical convergence, and Bessel statistical Cauchy. And, we have provided some theorems related to these concepts. We established key inclusion relations and related results that highlight the interconnections among these new concepts. Additionally, our introduction of revised versions of the first and second Korovkin-type approximation theorems, based on Bessel statistical convergence, represents a significant advancement in approximation theory. The empirical validation of our theorems through examples utilizing the classical Bernstein operator and Fejér convolution operators underscores the robustness and applicability of our proposed framework. Overall, our findings provide a more comprehensive and nuanced understanding of sequence behavior compared to existing theories.

The advancements detailed in this paper pave the way for further research and development in the field, offering a solid foundation for future investigations into Bessel-type convergence and approximation methods. For further studies, we suggest that some research papers can be prepared using our results; for instance:

- In recent years several versions of approximation theorems have been presented by several authors, for instance, in [30, 31], the authors proposed the notions of statistical convergence using deferred Nörlund means. These versions can be further expanded by applying the concepts of our study. As a result, new versions of approximation theorems can be introduced using deferred Nörlund Bessel statistical convergence.
- To explore additional new papers utilizing Bessel statistical convergence, we encourage readers to review various versions of approximation theorems found in [32–35]. This integration may yield refined results that not only extend the current understanding of approximation theorems but also open new avenues for research in mathematical analysis and its related disciplines.
- In [36], the authors presented the notion of summability means of Fourier series of arbitrary periodic functions, whereas in [37], the authors presented the notion of uniform convergence of Fourier series. We propose extending these concepts by incorporating the Bessel function within the framework of Fourier series.
