# Correction: Bessel statistical convergence: New concepts and applications in sequence theory

**DOI:** 10.1371/journal.pone.0329900

**Published:** 2025-08-05

**Authors:** Ibrahim S. Ibrahim, Majeed A. Yousif, Pshtiwan Othman Mohammed, Dumitru Baleanu, Ahmad Zeeshan, Mohamed Abdelwahed

The Funding statement for this article is incorrect. The correct Funding statement is as follows: Researchers Supporting Project number (RSP2024R136), King Saud University, Riyadh, Saudi Arabia.
